# Mathematical values in the processing of Chinese numeral classifiers and measure words

**DOI:** 10.1371/journal.pone.0185047

**Published:** 2017-09-19

**Authors:** One-Soon Her, Ying-Chun Chen, Nai-Shing Yen

**Affiliations:** 1 Graduate Institute of Linguistics, National Chengchi University, Taipei, Taiwan; 2 Research Center for Mind, Brain, and Learning, National Chengchi University, Taipei, Taiwan; 3 Department of Psychology, National Chengchi University, Taipei, Taiwan; University of Akron, UNITED STATES

## Abstract

A numeral classifier is required between a numeral and a noun in Chinese, which comes in two varieties, sortal classifer (C) and measural classifier (M), also known as ‘classifier’ and ‘measure word’, respectively. Cs categorize objects based on semantic attributes and Cs and Ms both denote quantity in terms of mathematical values. The aim of this study was to conduct a psycholinguistic experiment to examine whether participants process C/Ms based on their mathematical values with a semantic distance comparison task, where participants judged which of the two C/M phrases was semantically closer to the target C/M. Results showed that participants performed more accurately and faster for C/Ms with fixed values than the ones with variable values. These results demonstrated that mathematical values do play an important role in the processing of C/Ms. This study may thus shed light on the influence of the linguistic system of C/Ms on magnitude cognition.

## Introduction

Chinese is a numeral classifier language, where an element known as numeral classifier that denotes a unit is essential when a noun (N) is quantified by a numeral (Num). Numeral classifiers come in two varieties, sortal classifer (C) and measural classifier (M), also known as ‘classifier’ and ‘measure word’, respectively. In Table 1, *ben* and *ke* are classifiers (C); *xiang* (box) and *da* (dozen) are measure words (M). In this paper, the category of numeral classifiers is referred to as C/M.

**Table 1:** Examples of Chinese numeral classifiers and measure words.

**Numeral Classifier (C)**	**Measure Word (M)**
五 本 雜誌	五 箱 雜誌
*wu ben zazhi*	*wu xiang zazhi*
5 C magazine	5 M-box magazine
‘5 magazines’	‘5 boxes of magazines’
十 顆 蘋果	十 打 蘋果
*shi ke pingguo*	*shi da pingguo*
10 C apple	10 M-dozen apple
‘10 apples’	‘10 dozens of apples’

C and M converge in that they appear in the same grammatical position and are mutually exclusive [1–3]. However, C and M diverge in that Cs qualify the noun but Ms quantify the noun [4–6]. To be more specific, Cs categorise nouns by highlighting certain salient or inherent properties of the noun, while Ms denote the quantity of the entity of the noun [5,7]. For example, *ben* in Table 1 highlights the volume feature and can only be used for a bound copy of printed materials, such as a book or a magazine, whereas *xiang* means “a box of”, which carries new information indicating the quantity of the noun being quantified, since a box can contain different amounts of any object.

This convergence and divergence between C and M were reconciled by Her [8], where an innovative mathematical view was proposed to interpret the relation between Num and C/M as multiplication. The distinction between C and M is encoded precisely as follows: [Num X N] = [[Num × X] N], where X = C iff X = 1, otherwise X = M. In other words, X being the element required between Num and N, X is C if its inherent mathematical value is 1; otherwise, X is M. For example, in *shi ke pingguo* (ten C apple), *shi* (ten) and *ke* (C) form a multiplicative unit, i.e., (10×1); likewise, in *shi da pingguo* (ten M-dozen apple), *shi* (ten) and *da* (M-dozen) also form a multiplicative unit, i.e., (10×12). Under this view, C and M thus converge as the multiplicand of Num, the multiplier. C and M occupy exactly the same syntactic position and thus belong to a single syntactic category. Yet, C and M diverge in terms of their respective inherent values: C = 1, M ≠ 1, and thus constitute two distinct subcategories.

Note that a multiplicand 1 is unique in that it is the only identity element, or neutral element, in multiplication; 1 is thus redundant in the multiplicative equation. A multiplicand with any other value, numerical or non-numerical, is not redundant. This unique property of multiplicand 1 can explain why Cs may behave differently from Ms, in spite of C/M as a single syntactic category [8].

Her and Wu [9] further proposed a taxonomy of the magnitude values that C/Ms encode, along two dimensions: numerical vs. non-numerical and fixed vs. variable (See Table 2). While M_1_ and M_2_ both encode numerical values, the former has fixed values and the latter does not. Likewise, M_3_ and M_4_ both encode non-numerical values, but the former has fixed values and the latter does not. Thus, C, M_1_ and M_3_ encode fixed values, while M_2_ and M_4_ do not.

**Table 2:** Types of mathematical values denoted by C/Ms.

Numerical	Fixed	**n = 1** e.g., *ben* (本), *ke* (顆), *tiao* (條), *zhi* (隻)	C
		**n = 2** e.g., *duei* (pair 對); **n = 12** e.g., *da* (dozen 打)	M_1_
	Variable	**n > 1** e.g., *pai* (row 排), *bang* (gang 幫), *die* (stack 疊)	M_2_
Non- numerical	Fixed	e.g., *gongjin* (kilogram 公斤), *gongli* (kilometer 公里)	M_3_
	Variable	e.g., *di* (drop 滴), *dai* (bag 袋), *bei* (cup 杯)	M_4_

M_2_ and M_4_ are thus similar in that their values are vague and not fixed. The difference is that the vague value of a M_2_ is numerical, while that of a M_4_ is not. For example, the M_2_ *cuo* ‘small gang’ must take a count noun, e.g., *yi cuo qiangdao* ‘a small gang of bandits’, and must have a numerical value larger than one. Likewise, the M_2_ *bang* ‘gang’ in *yi bang qiangdao* ‘a gang of bandits’ must also have a numerical value larger than two. However, the typical number implied by *bang* ‘gang’ is larger than that implied by *cuo* ‘small gang’. In contrast to M_2_, the vague values denoted by M_4_ are not numerical and may be length, area, weight, volume, time, etc. For example, the M4 *di* ‘drop’, as in *yi di shui* ‘a drop of water’, refers to a vague volume of water in the shape of a teardrop. The M_4_ *tan* ‘puddle’, as in *yi tan shui* ‘a puddle of water’, though likewise referring to a vague volume of water, but in a random shape, has an implied value much bigger than that implied by *di* ‘drop’. Note, crucially, that the English counterparts of M_2_ and M_4_ are clearly nouns in terms of syntactic category. Yet, in Chinese, M_2_ and M_4_ are part of a distinctive syntactic category C/M, or numeral classifiers [8]. It is controversial whether the processing of Chinese numeral classifiers involves magnitude. The aim of our study was to address this issue.

However, as attractive as this theory may be, empirical evidence of the mathematical function of C/Ms was lacking. Thus, the aim of this study was to conduct a psycholinguistic experiment to examine whether participants process C/Ms based on their mathematical values as this multiplicative theory of C/M predicted they would. More specifically, the theory predicted that the difference between C/Ms with fixed values, i.e., C, M_1_, and M_3_, and those with non-fixed vague values, i.e., M_2_ and M_4_, would be more prominent than the difference between C/Ms with numerical values, i.e., C, M_1_, and M_2_, and those with non-numerical values, i.e., M_3_ and M_4_, for the simple reason that a M_2/4_ with a vague value cannot be coerced into having a rigid fixed value without an appropriate and robust discourse context, while a M_3/4_ with an inherent non-numerical value can be quite easily converted numerically to a smaller unit, e.g., *one kilo* into *one thousand grams*.

The most relevant previous study is Cui et al. [10], where a functional magnetic resonance imaging (fMRI) experiment compared the brain activities of processing classifiers with those of processing tool nouns, numbers, and dot arrays. Tool nouns are non-quantity words which refer to concrete objects used as tools, utensils, or instruments, e.g., *liandao* (sickle) and *laba* (trumpet). A semantic distance comparison task was used, where participants chose from two items the one that was semantically closer to the target item. For example, the target *fuzi* (axe) was presented on the top of the screen and participants had to judge whether *liandao* (sickle) or *niezi* (tweezers) which were displayed at the bottom of the screen was semantically closer to the target word *fuzi* (axe). Greater activation was found in the left middle frontal gyrus (MTG) and the left inferior frontal gyrus (IFG) instead of the right intraparietal sulcus (IPS) for processing classifiers and tool nouns than numbers and dot arrays. This result is rather unexpected under Her’s [8] theory, which predicts that brain activities of processing C/Ms should be more similar to those of processing numbers and dot arrays than to those of processing tool nouns. Given that some C/Ms (see Table 2), numbers, and dot arrays represent numerical magnitude, we expected that the processing of C/Ms, but not that of tool nouns, would elicit higher activations in the right IPS, which plays an important role in representation of numerical magnitude [11–12].

One possible critical reason why Cui et al. [10] did not find the IPS more activated for processing C/Ms than processing tool nouns is that their experimental materials of the so-called “classifiers” mixed up Cs and Ms and thus no distinction was made between Cs and Ms. Yet, as reviewed above, linguistic studies suggested that Cs differ significantly from Ms [e.g., 5–6]. Furthermore, the taxonomy proposed by Her and Wu [9] also categorizes the mathematical values of C/Ms along the dimension of [fixed vs. variable]. Presumably, C/Ms with a fixed value may be related to exact representation of numbers, while C/Ms that encode a variable value may be associated with approximation. We hypothesized that participants would choose the C/M option that had the same or closer value as that of the target C/M, when the values in question were all fixed. However, it was unclear how participants would process C/Ms with variable values.

Note also that Cui et al. [10] did not use complete [Num X N] phrases, e.g. *yi zhang haibao* (1 C_-flat_ poster, one poster), as stimuli in the semantic distance comparison task. Rather, they used [Num X] phrases, e.g. *yi zhang* (1 C_- flat_), in their study. Thus, the semantic context was not strictly confined in their study. Therefore, we replicated the paradigm by Cui et al. [10] but used a more appropriate set of stimuli, i.e. [Num X N] phrases, e.g. *yi zhang haibao* (1 C_- flat_ poster, one poster), to examine whether C/Ms were processed based on mathematical values.

We hypothesized that, first, participants would compare the mathematical values the C/Ms encode and select the one with the same or closer value to the target C/M, and, second, the accuracy of C/Ms with fixed values to be higher than that of C/Ms with variable values.

## Method

### Participants

Twenty individuals (16 females, 4 males, ages 20–28, mean age = 22.6 ± 2.06) were recruited from National Chengchi University. Participants were right-handed, had normal or corrected-to-normal vision. Their first language is Mandarin. They gave written informed consent to the study approved by the Research Ethics Committee of National Taiwan University and received NT$100.

### Stimuli and experimental design

We conducted a 2 × 2 within-subject design. The two independent variables were the numerical type (numerical: C, M_1_, and M_2_ vs. non-numerical:M_3_ and M_4_) and mathematical value type (fixed value: C, M_1_, and M_3_ vs. variable value: M_2_ and M_4_). There were thirteen C/Ms for each condition (see S1 Table). Each C/M repeated twice as the target C/M. For each trial, another two C/Ms from the same condition were selected to be paired with the target C/M. When three C/Ms phrases were paired together for a trial, two experimenters produced a reasonable noun for this set of C/Ms to confine the semantic contexts. One experimenter created these [Num X N] phrases and the other experimenter checked if all phrases were clear and understandable. For the phrases that were unclear, the two experimenter discussed and came up with another noun that better fit the set of C/Ms. The nouns were unrepeated throughout the experiment. Consequently, there were 104 sets of C/M phrases in total. Each condition included 26 trials. The target C/M phrases were composed of the number 1 and a C/M. The answer and distractor C/M phrases included the number 1, a C/M, and a noun. The answer and distractor C/M phrases differed in the C/M. The numeral enabled participants to process the C/M as a C/M, not a noun. By designing the answer/distractor phrase as a minimal pair, we strictly confined the semantic context for the C/M (Table 3). We recorded responses and reaction times (RT).

**Table 3:** Structure of the experimental stimuli with a sample set for each condition.

Stimuli type	Value type	Target C/M	C/M Option 1	C/M Option 2
Numerical	Fixed	一副 *yi fu* one set of (M_1_, n = 2)	**一對耳環 *yi dui erhuan* one pair of earrings (M**_**1**_**, n = 2)**	一只耳環 *yi zhi erhuan* one earring (C, n = 1)
	Variable	一隊 *yi dui* one team of (M_2_, n > 1)	一群殺手 *yi qun shashou* one group of killers (M_2_, n > 1)	一幫殺手 *yi bang shashou* one gang of killers (M_2_, n > 1)
Non-numerical	Fixed	一公斤 *yi gongjin* one kilo of (M_3_)	**一磅橡膠 *yi bang xiangjiao* one pound of rubber** (M_3_)	一噸橡膠 *yi dun xiangjiao* one ton of rubber (M_3_)
	Variable	一杯 *yi bei* one cup of (M_4_)	一罐咖啡 *yi guan kafei* one can of coffee (M_4_)	一瓶咖啡 *yi ping kafei* one bottle of coffee (M_4_)

There were 26 trials for each condition in the experiment. In each trial, there was a target C/M phrase, an answer C/M phrase, and a distractor C/M phrase. The target C/M phrases were composed of the number 1 and a C/M. The answer and distractor C/M phrases formed a minimal pair which included the number 1, a C/M, and a noun. The answer C/M phrases were indicated in bold in this table. Note that they were *not* presented in bold in the experiment.

### Procedure

**Fig 1:**
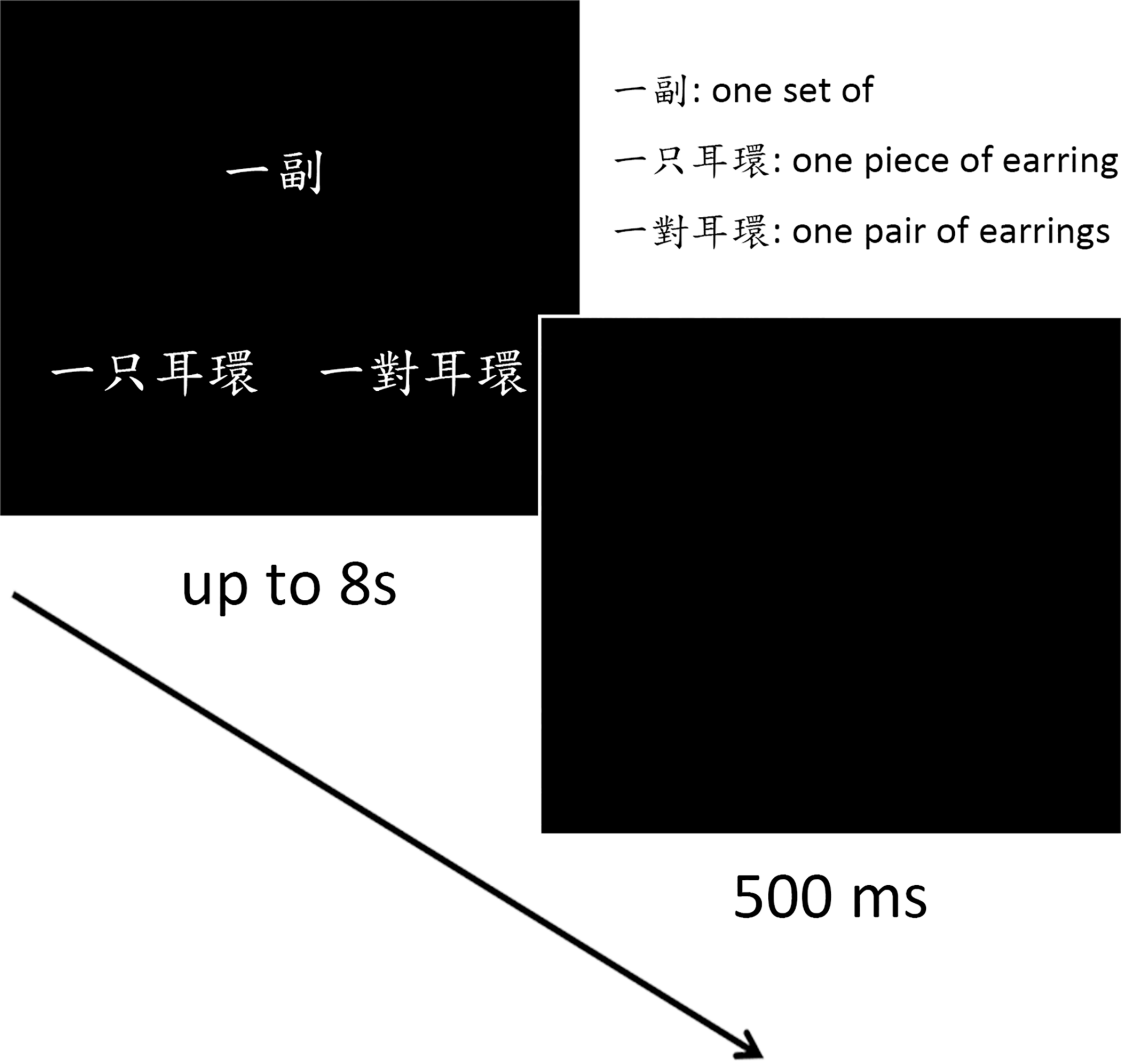
The experimental procedure. In each trial, participants saw three C/M phrases on the screen. They had to choose between the two C/M phrases at the bottom the one that was semantically closer to the target C/M phrase on the top. The C/M options were composed of minimal pairs which included an identical numeral, a classifier or a measure word, and an identical noun.

There were eight practice trials to ensure that participants fully understood the task. In each trial, participants saw three C/M phrases on the screen at the positions of the three end points of a triangle (Fig 1). They had to perform a semantic distance comparison task: which one of the two C/M phrases at the bottom was semantically closer to the target C/M phrase at the top. The positions of the answers and the distractors were randomized. Participants had up to 8 seconds (s) to respond. The inter-trial interval was 500 milliseconds (ms). There were 104 trials in total; each condition included 26 trials. The order of the trials was randomized. Right after the experiment, participants filled in a questionnaire to indicate their subjective mathematical values of the M_2_ and M_4_ used in the experiment. The questionnaire listed all C/Ms that appeared in the experiment in the form of [one C/M]. Take *yi fu* (one set of) for example, participants had to fill in the blank *yue ““ge* (around ““C) to indicate their subjective mathematical value. For non-numerical M_3-4_, participants had to fill in the blank to indicate around how much centimeter (length), square meter (area), gram (weight), and milliliter (volume) they think the M_3-4_ represented for different types of M_3-4_ respectively. For example, when participants saw *yi bei* (one cup of), they had to fill in the blank *yue ““haosheng* (around ““milliliter) to indicate their subjective mathematical value of *yi bei* (one cup of).

### Data analysis

The responses and RT were analysed in a two-way (numerical/ non-numerical × fixed/ variable values) repeated measures ANOVA. IBM SPSS 20.0 was used for the statistical analysis with the α value set at .05. Post-hoc analyses of the simple main effects were made by means of t-tests applying Bonferroni’s correction for multiple comparisons. We calculated the accuracy of M_2_ and M_4_ based on the subjective mathematical values reported by the participants individually (see S1 Table for the descriptive statistic reports). For each participant, we determined the correct answer of each trial according to the subjective mathematical values that they reported. Take the sample set of M_2_ in Table 3 for example, if a participant reported that his/her subjective mathematical values of *yi dui* (one team of), *yi qun* (one group of), and *yi bang* (one gang of) were 10, 20, and 30, respectively, the answer of this trial for this participant would be *yi qun shashou* (one group of killers) instead of *yi bang shashou* (one gang of killers), as 20 is closer to 10 than 30 is.

## Results

The mean (and standard deviation, SD) of accuracy and RT are shown in Table 4.

**Table 4:** The mean (and standard deviation) of accuracy and reaction times (RT) in experiment.

	Numerical C/M	Non-numerical C/M
Fixed value		
Accuracy (proportion correct)	0.734 (0.086)	0.744 (0.079)
RT (s)	2.446 (0.634)	2.511 (0.585)
Variable value		
Accuracy (proportion correct)	0.568 (0.130)	0.583 (0.124)
RT (s)	2.794 (0.708)	2.886 (0.666)

### Accuracy

The significant main effect of the numerical types was not significant, *F*_(1,19)_ = .227, *p* = .639, such that the accuracy of the numerical C, M_1_, and M_2_ and that of the non-numerical M_3_ and M_4_ were not significantly different. However, there was a significant main effect of the mathematical value types, such that the accuracy of C/Ms with fixed values was significantly higher than those with variable values, *F*_(1,19)_ = 68.298, *p* < .001. The accuracy of C and M_1_ was significantly higher than that of M_2_ (*p* < .001), and the accuracy of M_3_ was significantly higher than that of M_4_ (*p* < .001). There was no significant interaction effect between the numerical types and the mathematical value types, *F*_(1,19)_ = .013, *p* = .91 (Table 4).

### Reaction times

The significant main effect of the numerical types was not significant, *F*_(1,19)_ = 2.098, *p* = .164, such that the RT of numerical C/M_1-2_ and that of non-numerical M_3-4_ were not significantly different. However, the mathematical value types displayed a significant main effect, *F*_(1,19)_ = 37.726, *p* < .001, such that the participants responded faster while processing the C/M with fixed values compared with the C/M with variable values. The RT of C/M_1_ was significantly shorter than M_2_ (*p* = .001), and the RT of M_3_ was significantly shorter than M_4_ (*p* < .001). There was no interaction between the numerical types and the mathematical value types, *F*_(1,19)_ = .068, *p* = .797 (Table 4). In general, the pattern of reaction times under the four conditions was consistent with that of accuracy. The higher the accuracy, the shorter the RT.

## Discussion

The results showed that participants made semantic judgments based on the mathematical values of C/Ms, numerical or not, when the values were fixed rather than variable. They responded faster in processing C/Ms with a fixed value than a variable value. The mean accuracy of M_2_ (0.568) and M_4_ (0.583) with variable values was relatively low, even though the accuracy was calculated individually dependent on the subjective mathematical values reported by each participant. This was consistent with our prediction that C/Ms with fixed values are mathematically comparable, while C/Ms with variable values are too vague to be comparable.

It is still possible that participants represented a rough value that M_2_ or M_4_ encode and tried to compare. However, because of the variability of the values they encode, participants may not be able to represent these variable values exactly the same way every time. In other words, the subjective mathematical values may have fluctuated between the time of performing the semantic distance comparison task and filling in the post-experimental questionnaire. To modify this limitation in the current study, we suggest future studies ask participants to report their subjective mathematical values immediately after each trial.

It is worth noting that the mean subjective mathematical value of M_2_ ranged only from 5 to 18 and the variance was rather small (see S1 Table). This may make choosing between the two options of C/Ms difficult and result in fifty percent of chance to choose one of the two options of C/Ms. Even if the participants represented M_2_ as a mathematical value, the closeness of the two options of C/Ms may be too competitive to make a distinct difference. Furthermore, although the mean subjective mathematical value of M_4_ varied to a greater extent than M_2_ did, the variance was large. This indicates that there was a large individual difference of the subjective mathematical values of M_4_s. Future studies are suggested to use a complete sentence or story to confine the context to better control the semantic distance of C/Ms. Since behavioral responses could not answer whether participants processed C/Ms with variable values mathematically, future studies can further investigate the quantity processing of C/Ms that encode variable values using fMRI by examining the brain activations related to numerical representation such as the IPS [11–12].

One may argue that the quantification of *a gang of* might be different for killers and for hooligans and the quantification of *a litter of* might be different for mice than for cats. This indeed may be true for M_2_ and M_4_, which have non-fixed variable values. Yet, this possible noun-contingency effect was not a factor in the experiment, as all minimal pairs of [one M_2/4_ N] have exactly the same N. One may also suspect that the discriminability of these pairs might vary over nouns, e.g., the difference between *a team of salespeople* and *a gang of salespeople* might be different in not only magnitude but sign from the difference between *a team of killers* and *a gang of killers*. Again, this possible effect was not a factor in the experiment as no such cross-pair comparison was elicited and only within-pair comparison was required.

Not surprisingly, there was no significant difference between numerical and non-numerical C/Ms. One of the reasons may be that we adopted the semantic distance comparison task in this experiment. It is likely that participants converted the non-numerical C/M into the same unit to make a comparison. For example, when participants had to choose between *yi bang* (a pound) and *yi gongjin* (one kilo), they represented them as 453 grams and 1000 grams, whether exactly or approximately, to make the judgment. In other words, it was possible that due to the nature of the semantic distance comparison task which may require accurate quantity comparison, participants preferred representing C/Ms as a numerical value to perform the task in the current study. This may explain why we did not observe significant difference between numerical and non-numerical C/Ms. We suggest future studies use other tasks to further investigate whether the cognitive processing of numerical and non-numerical C/Ms are similar in spite of experimental paradigms. Future study may also use neuroimaging techniques to examine whether numerical and non-numerical C/Ms engage in a similar neural network.

Partially consistent with our hypothesis that C/Ms encode mathematical values, we found that participants did represent and compared the mathematical values of C/Ms with fixed values. If participants did not process them based on their mathematical values, the accuracy of numerical C/Ms would not have been above the chance level. However, it remains unclear how participants processed the C/Ms with variable values. Moreover, it is unknown whether participants processed non-numerical C/Ms in a numerical form to perform the semantic distance comparison task. Therefore, future studies are needed to further examine the cognitive processing of non-numerical C/Ms using other tasks. In general, our findings, in part, corroborated Her's [8] mathematical theory of C/M that C/Ms encode mathematical values by providing behavioral evidence of C/Ms with fixed mathematical values.

To our knowledge, this study was the first study providing evidence that showed Chinese C/Ms encode mathematical values. Participants represented and compared fixed mathematical values of C/Ms to make a semantic judgment. This psychological finding laid an empirical foundation supporting Her's [8] mathematical theory, where C and M converge as the multiplicand of Num but diverge in terms of their respective value: C = 1, M ≠ 1. We verified the notion that Cs encode 1 and Ms encode certain other mathematical values by showing that participants chose the C/M that had the same or closer value to the target C/M when the mathematical values were fixed in the semantic distance comparison task. Future psycholinguistic and neurolinguistic studies should further investigate whether the mathematical relation between Num and C/M is multiplication. In sum, findings in the current study implied that the linguistic system of C/Ms might influence magnitude cognition.

## Supporting information

S1 Dataset Mean accuracy and RT in the current study. (DOCX)

S1 Table Stimuli used in the experiment. The word frequency was obtained from the Digital Resources Center for Global Chinese Language Teaching and Learning by Cheng et al. [13].
(DOCX)

## References

[1] He J. Xiandai Hanyu Liangci Yanjiu [A Study of Measures in Modern Chinese]. Beijing: Beijing Language University Press; 2008.

[2] Hsieh ML. The internal structure of noun phrases in Chinese Crane Publishing Company; 2008.

[3] Her OS. Structure of classifiers and measure words: A lexical functional Account. Language and Linguistics. 2012 11 1; 13(6): 1211.

[4] Adams KL, Conklin NF. Toward a theory of natural classification. InAnnual Regional Meeting of the Chicago Linguistic Society 1973 4 13 (Vol. 9, pp. 1–10).

[5] Her OS, Hsieh CT. On the semantic distinction between classifiers and measure words in Chinese. Language and linguistics. 2010 3 1; 11(3): 527–51.

[6] Li X, Rothstein S. Measure readings of Mandarin classifier phrases and the particle de. Language and Linguistics. 2012 7 1; 13(4): 693–741.

[7] Tai J, Wang L. A Semantic Study of the Classifier Tiao. Journal of the Chinese Language Teachers Association. 1990; 25(1): 35–56.

[8] Her OS. Distinguishing classifiers and measure words: A mathematical perspective and implications. Lingua. 2012 11 30; 122(14): 1668–91.

[9] Her OS, Wu JS. Taxonomy of numeral classifiers and measure words: A formal semantic proposal. Under review with Linguistics. 2017.

[10] Cui J, Yu X, Yang H, Chen C, Liang P, Zhou X. Neural correlates of quantity processing of numeral classifiers. Neuropsychology. 2013 9; 27(5): 583–94. doi: 10.1037/a0033630 23937482 10.1037/a0033630

[11] Dehaene S, Piazza M, Pinel P, Cohen L. Three parietal circuits for number processing. Cognitive neuropsychology. 2003 5 1; 20(3–6): 487–506. 20957581 10.1080/02643290244000239

[12] Nieder A, Dehaene S. Representation of number in the brain. Annual review of neuroscience. 2009 7 21; 32: 185–208. doi: 10.1146/annurev.neuro.051508.135550 19400715 10.1146/annurev.neuro.051508.135550

[13] Cheng CC, Huang CR, Lo FJ, Tsai MC, Huang YC, Chen XY, et al. Digital Resources Center for Global Chinese Language Teaching and Learning. 2005 [cited 22 May 2017]. Available from: http://elearning.ling.sinica.edu.tw/index.html.

