# Supplementary material for: Mathematical values in the processing of Chinese numeral classifiers and measure words
Source: PLoS One. 2017 Sep 19;12(9):e0185047. doi: 10.1371/journal.pone.0185047 (PMC5605055; doi:10.1371/journal.pone.0185047)
Supplement: S1 Dataset — (DOCX) [file pone.0185047.s001.docx]

| Accuracy | | | | |
| --- | --- | --- | --- | --- |
|  | fixed | variable | fixed | variable |
|  | numerical | | non-numerical | |
| Subject | C+M1 | M2 | M3 | M4 |
| 1 | 69.2% | 73.7% | 69.2% | 42.3% |
| 2 | 69.2% | 62.5% | 61.5% | 70.0% |
| 3 | 57.7% | 59.1% | 65.4% | 32.0% |
| 4 | 73.1% | 50.0% | 80.8% | 57.7% |
| 5 | 57.7% | 25.0% | 73.1% | 58.3% |
| 6 | 76.9% | 50.0% | 80.8% | 71.4% |
| 7 | 76.9% | 75.0% | 76.9% | 58.3% |
| 8 | 76.9% | 54.5% | 69.2% | 64.0% |
| 9 | 76.9% | 75.0% | 68.0% | 77.8% |
| 10 | 73.1% | 62.5% | 65.4% | 63.6% |
| 11 | 69.2% | 50.0% | 92.3% | 73.9% |
| 12 | 73.1% | 50.0% | 69.2% | 58.3% |
| 13 | 92.3% | 68.8% | 76.9% | 54.2% |
| 14 | 84.6% | 62.5% | 76.9% | 43.5% |
| 15 | 76.9% | 44.4% | 80.8% | 70.8% |
| 16 | 80.8% | 38.9% | 69.2% | 66.7% |
| 17 | 76.0% | 43.8% | 69.2% | 50.0% |
| 18 | 73.1% | 60.0% | 73.1% | 65.4% |
| 19 | 57.7% | 66.7% | 84.6% | 45.8% |
| 20 | 76.9% | 63.6% | 84.6% | 42.3% |
| Mean | 73.4% | 56.8% | 74.4% | 58.3% |
| SD | 0.086 | 0.130 | 0.079 | 0.124 |
| SEM | 0.019 | 0.029 | 0.018 | 0.028 |

| RT | | | | |
| --- | --- | --- | --- | --- |
|  | fixed | variable | fixed | variable |
|  | numerical | | non-numerical | |
| Subject | C+M1 | M2 | M3 | M4 |
| 1 | 2.33 | 2.80 | 2.30 | 3.08 |
| 2 | 2.14 | 2.88 | 2.59 | 2.80 |
| 3 | 3.25 | 3.11 | 3.56 | 3.91 |
| 4 | 2.09 | 2.46 | 2.07 | 2.45 |
| 5 | 3.13 | 3.06 | 3.02 | 3.13 |
| 6 | 1.57 | 1.78 | 2.06 | 1.78 |
| 7 | 2.23 | 2.92 | 2.60 | 3.24 |
| 8 | 2.09 | 2.43 | 1.77 | 2.37 |
| 9 | 2.39 | 2.92 | 2.66 | 3.61 |
| 10 | 3.53 | 3.47 | 3.01 | 3.51 |
| 11 | 2.78 | 3.09 | 2.72 | 2.77 |
| 12 | 3.62 | 4.02 | 3.53 | 4.12 |
| 13 | 1.96 | 2.31 | 2.48 | 2.30 |
| 14 | 2.00 | 2.04 | 2.05 | 2.31 |
| 15 | 2.41 | 2.60 | 2.16 | 2.88 |
| 16 | 2.55 | 2.54 | 2.20 | 2.77 |
| 17 | 3.00 | 4.56 | 3.11 | 3.55 |
| 18 | 1.36 | 1.51 | 1.34 | 1.68 |
| 19 | 2.81 | 3.11 | 3.04 | 3.20 |
| 20 | 1.66 | 2.29 | 1.95 | 2.27 |
| Mean | 2.45 | 2.79 | 2.51 | 2.89 |
| SD | 0.63 | 0.71 | 0.58 | 0.67 |
| SEM | 0.14 | 0.16 | 0.13 | 0.15 |
