# Supplementary material for: Mathematical values in the processing of Chinese numeral classifiers and measure words
Source: PLoS One. 2017 Sep 19;12(9):e0185047. doi: 10.1371/journal.pone.0185047 (PMC5605055; doi:10.1371/journal.pone.0185047)
Supplement: S1 Table — The word frequency was obtained from the Digital Resources Center for Global Chinese Language Teaching and Learning by Cheng et al. [13]. (DOCX) [file pone.0185047.s002.docx]

| C | 一根 | 一員 | 一張 | 一只 | 一枝 |  |  |  |  |  |  |  |  |
| --- | --- | --- | --- | --- | --- | --- | --- | --- | --- | --- | --- | --- | --- |
|  | *yi gen* | *yi yuan* | *yi zhang* | *yi zhi* | *yi zhi* |  |  |  |  |  |  |  |  |
| Meaning | one stick of | unit for persons | one sheet of | one piece of | unit for something long-shaped |  |  |  |  |  |  |  |  |
| Frequency | 322 | 62 | 1017 | 97 | 72 |  |  |  |  |  |  |  |  |
| Strokes | 10 | 10 | 11 | 5 | 8 |  |  |  |  |  |  |  |  |
| M1 | 一打 | 一刀 | 一對 | 一副 | 一令 | 一旅 | 一雙 | 一營 |  |  |  |  |  |
|  | *yi da* | *yi dao* | *yi dui* | *yi fu* | *yi ling* | *yi lu* | *yi shuang* | *yi ying* |  |  |  |  |  |
| Meaning | one dozen | unit for paper | one pair of | one set of | unit for paper | one brigade of | one pair of | one battalion of |  |  |  |  |  |
| Frequency | 13 | 3 | 369 | 168 | 7 | 169 | 153 | 26 |  |  |  |  |  |
| Strokes | 5 | 2 | 14 | 11 | 5 | 10 | 18 | 17 |  |  |  |  |  |
| M2 | 一幫 | 一串 | 一疊 | 一隊 | 一行 | 一伙 | 一夥 | 一捆 | 一列 | 一群 | 一套 | 一窩 | 一組 |
|  | *yi bang* | *yi chuan* | *yi die* | *yi dui* | *yi hang* | *yi huo* | *yi huo* | *yi kun* | *yi lie* | *yi qun* | *yi tao* | *yi wo* | *yi zu* |
| Meaning | one gang of | one string of | one stack of | one team of | one file of | one group of | one group of | one bundle of | one row of | one group of | one set of | one litterof | one group of |
| Frequency | 25 | 52 | 25 | 282 | 139 | 6 | 29 | 1 | 50 | 581 | 970 | 9 | 441 |
| Strokes | 17 | 7 | 22 | 12 | 6 | 6 | 14 | 10 | 6 | 13 | 10 | 14 | 11 |
| Mean (and SD) of subjective mathematical value | 18.25  (2.88) | 6.55  (0.89) | 15.60  (2.44) | 14.05  (2.04) | 8.80  (0.68) | 9.25  (0.93) | 11.25  (1.46) | 13.00  (1.40) | 8.55  (0.63) | 10.88  (1.43) | 5.65  (0.85) | 14.55  (2.68) | 6.20  (0.73) |

| M3 | 一磅 | 一尺 | 一寸 | 一噸 | 一公尺 | 一公分 | 一公斤 | 一公里 | 一甲 | 一克 | 一兩 | 一畝 | 一坪 |
| --- | --- | --- | --- | --- | --- | --- | --- | --- | --- | --- | --- | --- | --- |
|  | *yi bang* | *yi chi* | *yi cun* | *yi dun* | *yi gongchi* | *yi gongfen* | *yi gongjin* | *yi gongli* | *yi jia* | *yi ke* | *yi liang* | *yi mu* | *yi ping* |
| Meaning | one pound | unit of length | unit of length | one ton | one meter | one centimeter | one kilo | one kilometer | unit of area | one gram | unit of weight | unit of area | unit of area |
| Frequency | 38 | 76 | 69 | 173 | 676 | 320 | 315 | 481 | 23 | 4 | 73 | 87 | 183 |
| Strokes | 15 | 4 | 3 | 16 | 8 | 8 | 8 | 11 | 5 | 7 | 8 | 10 | 8 |
| M4 | 一包 | 一杯 | 一袋 | 一缸 | 一罐 | 一鍋 | 一盒 | 一壺 | 一盤 | 一瓶 | 一桶 | 一碗 | 一箱 |
|  | *yi bao* | *yi bei* | *yi dai* | *yi gang* | *yi guan* | *yi guo* | *yi he* | *yi hu* | *yi pan* | *yi ping* | *yi tong* | *yi wan* | *yi xiang* |
| Meaning | one bag of | one cup of | one bag of | one urn of | one can of | one pot of | one box of | one kettle of | one plate of | one bottle of | one bucket of | one bowl of | one box of |
| Frequency | 97 | 227 | 17 | 6 | 37 | 14 | 32 | 15 | 162 | 136 | 55 | 99 | 45 |
| Strokes | 5 | 8 | 11 | 9 | 24 | 17 | 11 | 12 | 15 | 10 | 11 | 13 | 15 |
| Mean (and SD) of subjective mathematical value | 322.00  (39.11)  gram | 368.00  28.65  milliliter (ml) | 1061.50  (508.17)  gram | 518400.00  (499072.55)  milliliter (ml) | 454.50  (39.24)  milliliter (ml) | 3195.00  (737.79)  milliliter (ml) | 441.00  (91.48)  gram | 857.50  (130.85)  milliliter (ml) | 264.50  (42.89)  milliliter (ml) | 567.50  (23.58)  milliliter (ml) | 2875.00  (682.91)  milliliter (ml) | 315.00  (23.81)  milliliter (ml) | 2800.00  (568.52)  gram |
